# Soluble urokinase plasminogen activator receptor across the kidney–cardiometabolic continuum: a systematic review

**DOI:** 10.3389/fimmu.2026.1940345

**Published:** 2026-10-22

**Authors:** Qishun Wu, Zhiliang Yu, Xin Lin, Ling Yang, Zhangli Wu

**Affiliations:** Division of Nephrology, Department of Medicine, The Second Affiliated Hospital of Wannan Medical University, Wuhu, Anhui, China

**Keywords:** innate immunity, decision curve analysis, soluble urokinase-type plasminogen activator receptor, cardiovascular disease, sex differences, chronic kidney disease, assay standardization, risk stratification

## Abstract

**Background:**

Soluble urokinase plasminogen activator receptor (suPAR), a circulating marker of innate immune activation, has been proposed as a risk biomarker in chronic kidney disease (CKD) and related cardiometabolic conditions, but its clinical utility remains uncertain.

**Methods:**

We performed a systematic review according to PRISMA 2020 and the Synthesis Without Meta-analysis (SWiM) guideline, searching PubMed, Embase, Web of Science, Scopus, the Cochrane Library, medRxiv, ClinicalTrials.gov, and WHO ICTRP from inception to May 15, 2026. Two reviewers independently screened records, extracted data, and assessed quality using the Newcastle-Ottawa Scale, the JBI checklist, RoB 2, and QUADAS-2; certainty of evidence was rated with the Grading of Recommendations Assessment, Development and Evaluation (GRADE) framework.

**Results:**

Twenty studies comprising 31,416 participants were included. In adults with preserved kidney function, elevated suPAR predicted incident CKD (adjusted odds ratio (OR) 3.13 for the highest versus lowest quartile, 95% confidence interval (CI) 2.11–4.65) and end-stage kidney disease (ESKD) (adjusted hazard ratio (HR) 1.53 for the highest versus lowest tertile, 95% CI 1.10–2.12), but the gain in discrimination was modest (absolute C-statistic increase 0.027–0.08) and no study performed decision curve analysis (DCA). suPAR did not distinguish focal segmental glomerulosclerosis (FSGS) from other glomerular diseases, and prognostic evidence in FSGS rested on a single moderate-quality study. In pediatric CKD and in metabolic cohorts, suPAR lost independent association after adjustment for baseline kidney function or inflammatory markers. Associations with cardiovascular outcomes were directionally consistent but observational and of low certainty. No study reported sex-stratified analyses.

**Conclusions:**

Routine clinical measurement of suPAR is not currently supported; assay harmonization, sex-disaggregated analyses, prospective validation with decision curve analysis, and trials of suPAR-targeted therapy are needed first.

**Systematic Review Registration:**

https://www.crd.york.ac.uk/PROSPERO/view/CRD420261320503, identifier CRD420261320503.

## 1 Introduction

Chronic kidney disease (CKD) affects roughly 9% of the global population and carries a heavy burden of cardiovascular and metabolic complications (1). Routine screening relies on estimated glomerular filtration rate (eGFR) and albuminuria, both of which capture established injury rather than future risk. Soluble urokinase plasminogen activator receptor (suPAR), the circulating form of the glycosylphosphatidylinositol-anchored urokinase receptor (uPAR), has attracted attention as a candidate marker that rises before functional decline becomes apparent.

uPAR is expressed on activated immune cells, endothelial cells, and podocytes. Inflammatory stimulation triggers proteolytic cleavage of membrane uPAR, releasing intact suPAR (domains I–II–III, approximately 55–60 kDa) and smaller fragments (domains II–III, 20–50 kDa) into the circulation. Commercial assays differ in their affinity for these isoforms, a technical point with direct consequences for cross-study comparability. In animal models, suPAR activates αvβ3 integrins on podocytes and produces foot process effacement and proteinuria (**Figure 1**). Whether suPAR plays the same causal role in human disease, or instead marks underlying inflammation, remains unresolved. Elevated suPAR has been linked to incident CKD, faster eGFR decline, and adverse cardiovascular outcomes, but the literature is scattered across disease areas, and negative studies have challenged its specificity for focal segmental glomerulosclerosis (FSGS).

**Figure 1:**
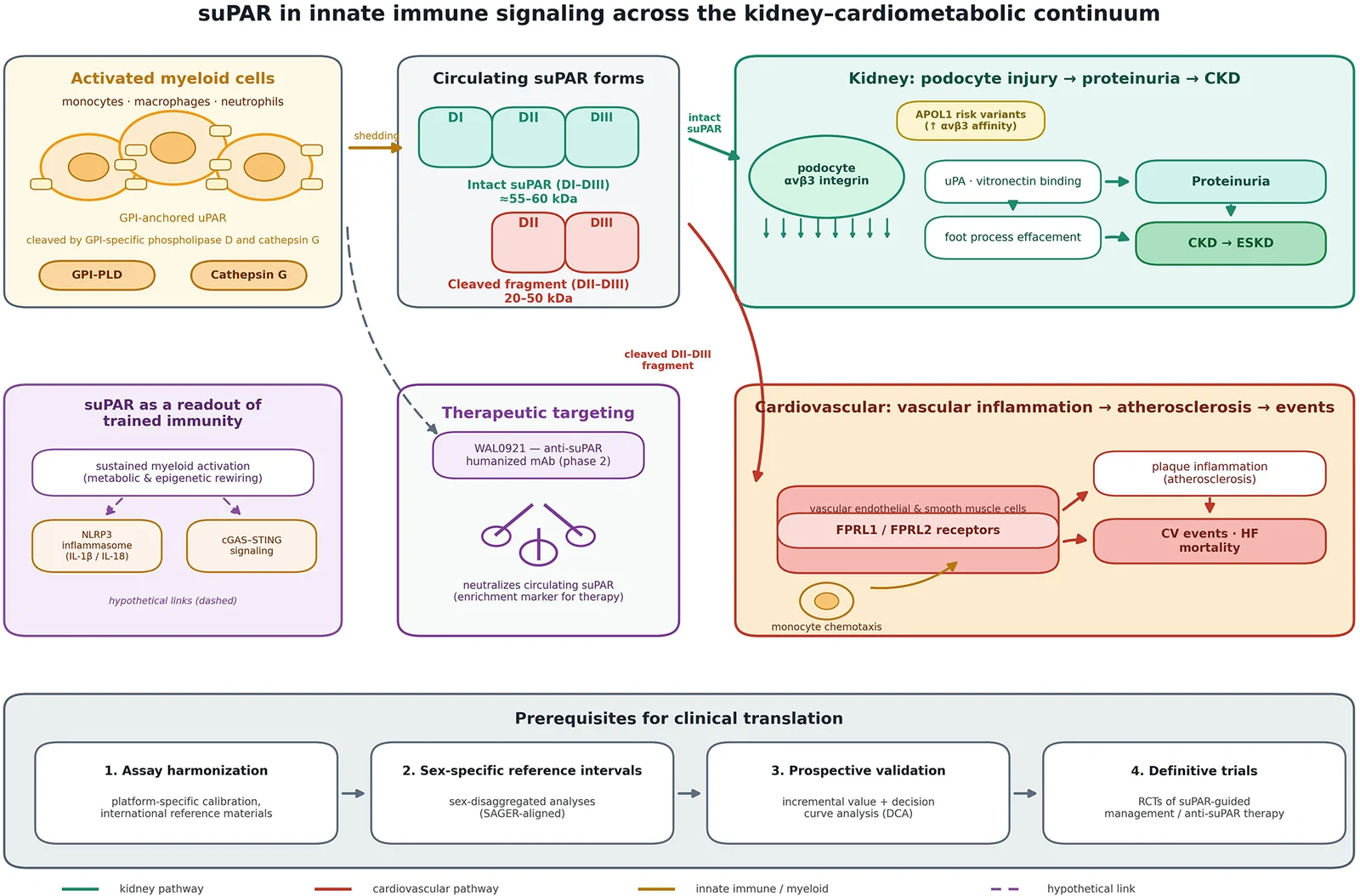
suPAR in innate immune signaling across the kidney–cardiometabolic continuum (redrawn). Activated myeloid cells (monocytes, macrophages, and neutrophils) release suPAR through GPI-specific phospholipase D and protease (cathepsin G) cleavage. Intact suPAR (DI–DII–DIII) binds urokinase-type plasminogen activator (uPA) and vitronectin and activates podocyte αvβ3 integrins, causing foot process effacement and proteinuria; APOL1 high-risk variants potentiate this interaction. The cleaved DII–DIII fragment engages formyl peptide receptor-like 1/2 (FPRL1/2) on vascular endothelial and smooth muscle cells, promoting monocyte chemotaxis and atherosclerotic inflammation. suPAR is also a candidate readout of trained immunity; links to NLRP3 inflammasome and cGAS–STING signaling (dashed arrows) remain hypothetical because direct evidence is lacking. WAL0921, an anti-suPAR monoclonal antibody in phase 2 development, neutralizes circulating suPAR. The lower band summarizes the prerequisites for clinical translation. GPI, glycosylphosphatidylinositol; suPAR, soluble urokinase plasminogen activator receptor.

Previous reviews each covered a narrow slice of this field: a meta-analysis of suPAR in CKD up to 2019 (2), kidney function decline in people without CKD (3), acute kidney injury (4), cardiovascular disease (5), and a cross-organ narrative review (6). The present review covers the full kidney–cardiometabolic spectrum in a single evidence synthesis. We catalog assay heterogeneity and its consequences for clinical translation, give equal weight to positive and negative findings, and examine the lack of sex-stratified evidence as a methodological gap, in line with the Sex and Gender Equity in Research (SAGER) guidelines (7).

## 2 Materials and methods

### 2.1 Protocol and registration

The review followed the PRISMA 2020 statement (8) and theSynthesis Without Meta-analysis (SWiM) reporting guideline (9). The protocol was registered with the International Prospective Register of Systematic Reviews (PROSPERO) (CRD420261320503) on February 17, 2026. The initial database search was run on January 15, 2026, before registration; screening, data extraction, and analysis began only after registration, and the analysis plan was not modified in light of emerging results. The final search update was performed on May 15, 2026. On March 5, 2026, we amended the search strategy after identifying a study from the Chinese Cohort Study of Chronic Kidney Disease (C-STRIDE) (10) that the original strategy had missed because the publication used the term “urokinase-type plasminogen activator receptor” without the abbreviation suPAR; all databases were re-run on March 6, 2026. Backward and forward citation chasing (March 10–15, 2026) yielded 23 further records, none of which was eligible.Complete search strategies are provided in **Supplementary Material 1**.

### 2.2 Search strategy

The strategy was developed with a medical librarian and peer-reviewed using the Peer Review ofElectronic Search Strategies (PRESS) checklist (11). Wesearched PubMed, Embase, Web of Science, Scopus, the Cochrane Library, and medRxiv, plus ClinicalTrials.gov, WHO ICTRP, and Google Scholar, from inception to May 15, 2026, combining suPAR-related free-text and MeSH terms with disease-specific keywords (**Supplementary Material 1**). Only English-language publications were eligible; Chinese-language databases were not searched, a restriction whose potential impact we address in the limitations.

### 2.3 Eligibility criteria

We included studies that (i) examined suPAR in CKD, glomerular disease, or cardiometabolic conditions with explicit clinical outcomes; (ii) reported original quantitative suPAR data in humans; and (iii) were peer-reviewed English-language articles, or medRxiv preprints later published in peer-reviewed form. We excluded reviews and meta-analyses, studies without quantitative suPAR data or clear clinical outcomes, studies with insufficient methodological detail for quality assessment, animal or *in vitro* work without clinical data, and, for overlapping datasets, all but the largest or most recent report.

The review question was structured according to Population, Intervention/Exposure, Comparator, Outcome, and Study design (PICOS): Population—adults or children with CKD, glomerular disease, or cardiometabolic conditions; Intervention/Exposure—circulating suPAR measured by any quantitative assay; Comparator—lower suPAR levels or reference ranges; Outcomes—incident CKD, CKD progression, ESKD, cardiovascular events, mortality, or metabolic complications; Study design—prospective or retrospective cohort, cross-sectional, or randomized studies with biomarker endpoints.

### 2.4 Study selection and quality assessment

Of 1,489 records, 1,052 duplicates were removed and 437 unique records screened. Title andabstract screening excluded 278 records. Of 159 full texts assessed, 139 were excluded (72 reviewsor meta-analyses, 35 without quantitative suPAR data, 18 overlapping datasets, 8 with insufficient methodological detail, 5 preprints without subsequent peer-reviewed publication, and 1 without relevant outcomes). One study was added through the amended search. A study with a Newcastle-Ottawa Scale score of 3/9 was retained in the review but flagged as high risk of bias in all analyses; sensitivity analyses excluding this study are presented in **Supplementary Material 5**. The final sample comprised 20 studies; the PRISMA 2020 flow diagram and the list ofexcluded studies with reasons are provided in **Supplementary Material 2**.

Cohort studies were assessed with the Newcastle-Ottawa Scale (NOS) (12), cross-sectional studies with the JBI Critical Appraisal Checklist, therandomized substudy with RoB 2 (13), and diagnostic accuracy analyses with QUADAS-2 (14). Two reviewers (Q.W., Z.Y.) assessed each study independently, with disagreements resolved by consensus; inter-rater agreement was substantial to almost perfect (Cohen’s κ 0.79–0.91). On the NOS, scores ≥7/9 were rated low risk of bias, 4–6 moderate, and <4 high; for the JBI checklist the corresponding cut-offs were ≥7/8, 5–6, and <5. Sixteen of the 19 observational studies were rated low risk and three moderate; the randomized component of Huet et al. (15) was rated low risk across all RoB 2 domains. Full assessments appear in **Supplementary Material 3**.

We acknowledge that the Newcastle-Ottawa Scale has known limitations for biomarker studies. Its broad domains may not fully capture exposure measurement drift during prolonged biobank storage, unmeasured confounding (e.g., socioeconomic status, inflammatory markers not adjusted for), or outcome misclassification. A domain-based tool specifically designed for observational biomarker studies, such as the Risk of Bias in Non-randomized Studies of Exposures (ROBINS-E), would provide a more rigorous and informative appraisal. In the present review, we retained NOS as the primary assessment tool to maintain consistency with the existing suPAR literature and prior biomarker reviews; however, we explicitly recognize this as a methodological limitation and recommend that future updates of this review employ ROBINS-E or similar domain-based instruments to enhance the robustness of quality assessment.

### 2.5 Data extraction

Two reviewers (Q.W., Z.Y.) extracted data independently using a predefined form, with a thirdreviewer (X.L.) adjudicating disagreements; the form template and author contact log are provided in**Supplementary Material 4**. Two of the 20 studies did not report sex distribution; for Luo et al. (16) these data were unavailable from the authors on request. Nostudy reported sex-stratified analyses or tested sex-by-suPAR interactions. We therefore emailed all 20 corresponding authors (two attempts, 14 days apart, between February 20 and April 15, 2026) requesting sex-disaggregated data; seven authors (35.0%) responded, none with usable data (**Supplementary Material 4**).

### 2.6 Synthesis and certainty of evidence

Given the heterogeneity of designs, populations, assays, outcome definitions, and effect metrics,we did not pool effect estimates; a narrative synthesis was performed following SWiM (9), with studies grouped by disease domain. Forest plots of multivariable-adjusted estimates are presented for each domain, and leave-one-out sensitivity analyses examined the influence of individual studies (**Supplementary Material 5**).

Meta-analysis was not performed for three reasons: suPAR exposure metrics (quartiles, tertiles, per-standard-deviation increments, or per-doubling) were not harmonized across studies; commercial assays detect different suPAR fragments with substantially different absolute concentrations; and outcome definitions varied widely (incident CKD, eGFR decline >30%, ESKD, cardiovascular events, and all-cause mortality). Pooling non-commensurable effect scales and non-equivalent endpoints would have produced clinically uninterpretable summary estimates, so narrative synthesis following SWiM was the only methodologically defensible approach.

Certainty of evidence was rated with GRADE for prognostic studies (17, 18), in which observational evidence starts at high certainty and is downgraded across five domains (risk of bias, inconsistency, indirectness, imprecision, publication bias) or upgraded for large effects, dose–response gradients, or opposing plausible confounding. Domain-level ratings are shown in **Supplementary Figures S5** and **S6**.

## 3 Results

### 3.1 Study characteristics

**Table 1** summarizes the 20 included studies (31,416 participants). Fourteen were prospective cohort studies, five cross-sectional, and one a prospective cohort with an embedded randomized substudy of colchicine. Sample sizes ranged from 60 to 6,576 (median 1,079). Thirteen studies were conducted in Europe, five in North America, one in China, and one in Japan. Mean or median age ranged from 12 to 77 years; among the 18 studies reporting sex distribution, women made up 27–56% of participants. suPAR was measured with the suPARnostic ELISA (ViroGates) in 14 studies, the R&D Systems ELISA in four, and both platforms in two; samples were serum in 11 studies, plasma in eight, and both in one. Reported median suPAR concentrations ranged from 2.4 ng/mL in suspected myocardial infarction to 11.81 ng/mL in acute heart failure (**Table 2**).

**Table 1:** Characteristics of the 20 included studies.

Study, year, country	Design	N	Population	Female (%)	Follow-up	suPAR assay	Sample	Quality score
Hayek et al., 2015, USA (19)	Prospective cohort	3,683	Emory CV Biobank	35	3.7 years	suPARnostic	Plasma	8/9 (NOS)
Schulz et al., 2017, Sweden (20)	Prospective cohort	4,529	Malmö Diet and Cancer Study	56	16 years	suPARnostic	Serum	8/9 (NOS)
Luo et al., 2018, USA (16)	Prospective cohort	1,910	CRIC (African American CKD)	NR†	NR	suPARnostic	Plasma	8/9 (NOS)
Lv et al., 2020, China (10)	Prospective cohort	2,391	C-STRIDE (Chinese CKD)	38	54.8 months	suPARnostic	Serum	8/9 (NOS)
Drechsler et al., 2017, Germany (21)	Prospective cohort	1,145	Diabetes on hemodialysis (4D)	42	NR	suPARnostic	Plasma	7/9 (NOS)
Abraham et al., 2021, USA (22)	Prospective cohort	586	CKiD (pediatric CKD)	38	NR	suPARnostic	Plasma	8/9 (NOS)
Meijers et al., 2014, Belgium (23)	Cross-sectional	78	Adults with glomerular disease	42	N/A	suPARnostic	Serum	7/8 (JBI)
Spinale et al., 2015, USA (24)	Prospective cohort	241	NEPTUNE (proteinuric GN)	46	NR	suPARnostic	Serum	8/9 (NOS)
Harita et al., 2014, Japan (25)	Cross-sectional	60	Children with FSGS	NR	N/A	R&D Systems	Serum	7/8 (JBI)
Winnicki et al., 2019, Austria (26)	Cross-sectional	146	Adults with glomerular diseases	45	N/A	suPARnostic + R&D	Plasma	5/8 (JBI)
Hayek et al., 2023, USA (27)	Prospective cohort	3,437	Coronary angiography patients	35	NR	suPARnostic	Plasma	8/9 (NOS)
Koller et al., 2017, Austria (28)	Prospective cohort	319	Chronic heart failure	27	NR	suPARnostic	Serum	8/9 (NOS)
Eugen-Olsen et al., 2010, Denmark (29)	Prospective cohort	6,576	General population	53	12.3 years	suPARnostic	Plasma	8/9 (NOS)
Sorensen et al., 2019, Germany (30)	Prospective cohort	1,116	Suspected myocardial infarction	35	1.0 year	suPARnostic	Plasma	8/9 (NOS)
Lyngbaek et al., 2012, Denmark (31)	Prospective cohort	1,042	STEMI post-PCI	27	3.8 years	suPARnostic	Plasma	8/9 (NOS)
Huet et al., 2021, France (15)	Prospective cohort + RCT	95	Acute heart failure	42	1 month/1 year	suPARnostic	Plasma	8/9 (NOS)
Dupuy et al., 2021, France (32)	Prospective cohort	182	Chronic heart failure	27	80 months	suPARnostic	Serum	8/9 (NOS)
Theilade et al., 2015, Denmark (33)	Cross-sectional	667	Type 1 diabetes	45	N/A	suPARnostic	Serum	7/8 (JBI)
Guthoff et al., 2017, Germany (34)	Prospective cohort	1,363	At risk for type 2 diabetes	48	NR	suPARnostic	Serum	8/9 (NOS)
Heraclides et al., 2013, Denmark (35)	Prospective cohort	1,850	Overweight, impaired glucose regulation	52	NR	suPARnostic	Plasma	8/9 (NOS)

NR, not reported; NOS, Newcastle-Ottawa Scale; JBI, Joanna Briggs Institute; CKD, chronic kidney disease; CRIC, Chronic Renal Insufficiency Cohort; C-STRIDE, Chinese Cohort Study of Chronic Kidney Disease; CKiD, Chronic Kidney Disease in Children; NEPTUNE, Nephrotic Syndrome Study Network; GN, glomerulonephritis; FSGS, focal segmental glomerulosclerosis; STEMI, ST-segment elevation myocardial infarction; PCI, percutaneous coronary intervention; RCT, randomized controlled trial. †Not reported in the manuscript; data unavailable upon author request.

**Table 2:** Reported suPAR levels by disease context.

Disease context	Study	N	Assay	Sample	Median suPAR (ng/mL)	Range
Preserved kidney function	Hayek et al., 2015	1,335	suPARnostic	Plasma	2.91	1.80–4.80
General population	Eugen-Olsen et al., 2010	6,576	suPARnostic	Plasma	2.44	1.80–3.40
Suspected MI	Sorensen et al., 2019	1,116	suPARnostic	Plasma	2.40	1.60–3.80
STEMI post-PCI	Lyngbaek et al., 2012	1,042	suPARnostic	Plasma	2.60	1.80–4.20
Chronic heart failure	Koller et al., 2017	319	suPARnostic	Serum	4.20	2.50–7.80
Acute heart failure	Huet et al., 2021	95	suPARnostic	Plasma	11.81	5.20–28.40
Type 1 diabetes	Theilade et al., 2015	667	suPARnostic	Serum	2.80	1.90–4.50
Pediatric CKD	Abraham et al., 2021	586	suPARnostic	Plasma	3.20	1.50–8.60
Chinese CKD	Lv et al., 2020	2,391	suPARnostic	Serum	3.20	1.80–6.40
FSGS (adults)	Winnicki et al., 2019	146	suPARnostic	Serum	4.80	2.10–12.50

The very high values observed in acute heart failure (median 11.81 ng/mL) should be interpreted with caution: impaired glomerular filtration reduces the renal clearance of cleaved suPAR fragments, and acute cardiometabolic distress often compromises kidney perfusion, so elevated circulating levels likely reflect reduced clearance as well as increased production.

### 3.2 suPAR in chronic kidney disease

Six studies examined CKD populations (**Table 3A**; **Supplementary Table S1**; **Supplementary Figure S1**). Effect directions were consistently positive in adults; the only null result came from the pediatric cohort.

**Table 3:** Key multivariable-adjusted effect estimates by disease domain.

A. CKD domain				
Study	Population	N	Outcome	Effect estimate (95% CI)
Hayek et al., 2015 (19)	eGFR ≥60 mL/min/1.73 m (2)	1,335	Incident CKD	OR 3.13 (2.11–4.65), Q4 vs Q1
Schulz et al., 2017 (20)	General population	4,529	eGFR decline >30%	HR 1.3 (1.1–1.5) per SD
Luo et al., 2018 (16)	African American CKD	1,910	eGFR decline	Independent association; higher suPAR with two APOL1 risk variants
Lv et al., 2020 (10)	Chinese CKD	2,391	ESKD	HR 1.53 (1.10–2.12), T3 vs T1
Drechsler et al., 2017 (21)	Diabetes on hemodialysis	1,145	All-cause mortality	HR 1.45 (1.15–1.84) per SD
Abraham et al., 2021 (22)	Pediatric CKD	586	CKD progression	HR 1.12 (0.89–1.41)
B. FSGS domain				
Meijers et al., 2014 (23)	Adults with glomerular disease	78	FSGS diagnosis	No discrimination from other GN
Spinale et al., 2015 (24)	Proteinuric GN (NEPTUNE)	241	FSGS diagnosis	No difference in suPAR change after adjustment
Harita et al., 2014 (25)	Children with FSGS	60	Elevated suPAR	Explained by GFR, not FSGS-specific pathology
Winnicki et al., 2019 (26)	Adults with glomerular diseases	146	ESKD progression	HR 2.77 (1.45–5.29)
C. Cardiovascular domain				
Hayek et al., 2023 (27)	Coronary angiography	3,437	All-cause death	HR 2.30 (1.90–2.77) per doubling
Koller et al., 2017 (28)	Chronic heart failure	319	Mortality	HR 1.38 (1.04–1.83) per SD
Eugen-Olsen et al., 2010 (29)	General population	6,576	Incident CVD	HR 1.42 (1.18–1.71) per SD
Sorensen et al., 2019 (30)	Suspected MI	1,116	All-cause mortality	Independent predictor over 1 year
Lyngbaek et al., 2012 (31)	STEMI post-PCI	1,042	Recurrent MI/mortality	Independent predictor over 3.8 years
Huet et al., 2021 (15)	Acute heart failure	95	1-month and 1-year mortality	Independent predictor
Dupuy et al., 2021 (32)	Chronic heart failure	182	Long-term mortality	Improved reclassification beyond MAGGIC
D. Metabolic domain				
Theilade et al., 2015 (33)	Type 1 diabetes	667	Diabetic nephropathy	OR 2.14 (1.45–3.16)
Guthoff et al., 2017 (34)	At risk for type 2 diabetes	1,363	Microalbuminuria	HR 2.3 (1.4–3.8), Q4 vs Q1
Heraclides et al., 2013 (35)	Overweight, impaired glucose regulation	1,850	Incident type 2 diabetes	HR 1.06 (0.97–1.16) after inflammation adjustment

In the Emory Cardiovascular Biobank (3,683 patients), Hayek et al. (19) found that higher baseline suPAR tracked with greater eGFR decline over a median of 3.7 years. Among 1,335 participants with baseline eGFR ≥60 mL/min/1.73 m^2^, the highest suPAR quartile carried a 3.13-fold risk of incident CKD versus the lowest quartile (95% CI 2.11–4.65), adjusted for demographics, comorbidities, and baseline eGFR. Adding suPAR to traditional risk factors raised the C-statistic for incident CKD by 0.08 at 1 year and 0.03 at 5 years, with net reclassification improvement (NRI) of 0.40 and 0.34, respectively. In the population-based Malmö Diet and Cancer Study (4,529 participants, median follow-up 16 years), each standard deviation (SD) increment in suPAR was associated with a 1.3-fold risk of eGFR decline >30% (95% CI 1.1–1.5) (20). Among 1,910 African American participants with CKD in the Chronic Renal Insufficiency Cohort, suPAR was higher in carriers of two APOL1 risk variants and independently associated with eGFR decline (16). In C-STRIDE (2,391 Chinese patients with CKD), the highest suPAR tertile (≥3.904 ng/mL) carried a 1.53-fold risk of end-stage kidney disease (ESKD) versus the lowest tertile (95% CI 1.10–2.12) after full adjustment, with a stronger association in glomerulonephritis (GN) (HR 1.61, 95% CI 1.03–2.53) than in diabetic or other etiologies (10). In 1,145 patients with diabetes on hemodialysis, each SD increase in suPAR was associated with all-cause mortality (HR 1.45, 95% CI 1.15–1.84) and cardiovascular events (HR 1.38, 95% CI 1.09–1.75) (21).

The exception was the pediatric Chronic Kidney Disease in Children (CKiD) study (586 children): after adjustment for eGFR and proteinuria, suPAR no longer predicted CKD progression (adjusted HR 1.12, 95% CI 0.89–1.41), consistent with suPAR reflecting reduced renal clearance rather than independent risk in mixed-etiology pediatric CKD (22).

GRADE certainty for incident CKD in preserved kidney function was moderate; certainty for progression of established CKD was low, and for pediatric CKD progression was very low (**Table 4**).

**Table 4:** GRADE summary of findings.

Outcome	Studies (n)	Participants (n)	Certainty	Main reasons for downgrading
Incident CKD in preserved kidney function	2	5,864	Moderate	Inconsistency (cut-offs, metrics); indirectness (selected populations)
Progression of established CKD	3	5,446	Low	Inconsistency (populations, assays, outcomes); indirectness
CKD progression in children	1	586	Very low	Single study; imprecision (CI crosses null); indirectness
FSGS diagnosis	3	379	Very low	Cross-sectional designs; small samples; predominantly null findings
FSGS prognosis (ESKD)	1	146	Very low	Single moderate-quality study; very small sample
Cardiovascular events and mortality	4	12,171	Low	Observational designs; heterogeneous phenotypes; selected populations
Heart failure outcomes	3	596	Low	Small samples; HF-specific populations
Microvascular complications in diabetes	2	2,030	Low	Cross-sectional component; imprecision
Incident type 2 diabetes	1	1,850	Very low	Single study; wide CI; selected population

### 3.3 suPAR as a diagnostic biomarker in FSGS

Three cross-sectional studies asked whether suPAR distinguishes FSGS from other glomerular diseases (**Table 3B**; **Supplementary Table S2**; **Supplementary Figure S2**). Meijers et al. (23) found no discrimination in 78 adults. In the Nephrotic Syndrome Study Network (NEPTUNE, 241 participants), changes in suPAR over time did not differ between FSGS and other diagnoses after adjustment (24). In 60 children with FSGS, reduced glomerular filtration, rather than FSGS-specific pathology, accounted for elevated circulating suPAR (25). Current evidence does not support suPAR as a diagnostic marker for FSGS.

### 3.4 suPAR as a prognostic biomarker in FSGS

A single study supports prognostic value. Winnicki et al. (26) compared the two commercial ELISAs in 146 adults with glomerular diseases:primary FSGS showed higher suPAR than other glomerulonephritides, and higher suPAR predicted progression to ESKD (HR 2.77, 95% CI 1.45–5.29). The optimal cut-off differed almost twofold between platforms (suPARnostic 4.644 ng/mL; R&D Systems 2.789 ng/mL). This study was of moderate quality (JBI 5/8), and leave-one-out analysis showed that removing it eliminated the only positive prognostic evidence in FSGS (**Supplementary Material 5**). Independent replication is required before any clinical use.

### 3.5 suPAR in cardiovascular disease

Seven studies addressed cardiovascular outcomes, comprising four general cardiovascular/atherosclerotic cohorts and three heart failure cohorts (**Table 3C**; **Supplementary Figure S3**); associations were uniformly positive. In 3,437 patients undergoing coronary angiography, each doubling of suPAR was associated with all-cause death (adjusted HR 2.30, 95% CI 1.90–2.77), cardiovascular death (HR 2.33, 95% CI 1.81–2.99), and heart failure hospitalization (HR 1.96, 95% CI 1.06–3.62), with an absolute C-statistic gain of **0.027** beyond B-type natriuretic peptide (27). In 319 patients with chronic heart failure, suPAR predicted mortality (HR 1.38 per SD, 95% CI 1.04–1.83) and improved risk reclassification (28). In 6,576 Danish adults followed for a median of 12.3 years, each SD increment was associated with incident cardiovascular disease (HR 1.42, 95% CI 1.18–1.71), incident diabetes (HR 1.25, 95% CI 1.08–1.45), and all-cause mortality (HR 1.56, 95% CI 1.35–1.80) (29). Consistent mortality associations were also reported in suspected myocardial infarction (1,116 patients) (30), ST-segment elevation myocardial infarction after primary percutaneous coronary intervention (1,042 patients) (31), acute heart failure (95 patients) (15), and chronic heart failure, where suPAR improved long-term risk reclassification beyond the Meta-Analysis Global Group in Chronic Heart Failure (MAGGIC) score (182 patients) (32).

GRADE certainty for cardiovascular events and mortality was low, as was certainty for heart failure outcomes (**Table 4**).

### 3.6 suPAR in metabolic disease

Three studies examined diabetes and metabolic risk (**Table 3D**; **Supplementary Figure S4**). In 667 adults with type 1 diabetes, elevated suPAR was associated with diabetic nephropathy (OR 2.14, 95% CI 1.45–3.16), retinopathy (OR 1.78, 95% CI 1.23–2.57), and cardiovascular autonomic neuropathy (OR 1.65, 95% CI 1.12–2.43) (33). In 1,363 individuals at risk for type 2 diabetes, the highest suPAR quartile carried a 2.3-fold risk of incident microalbuminuria (95% CI 1.4–3.8) (34). In contrast, in 1,850 overweight individuals with impaired glucose regulation, the association with incident type 2 diabetes disappeared after adjustment for C-reactive protein and interleukin-6 (HR 1.06, 95% CI 0.97–1.16), suggesting that suPAR marks the underlying inflammation rather than mediating diabetes risk (35).

GRADE certainty for microvascular complications in diabetes was low, and for incident type 2 diabetes was very low (**Table 4**).

### 3.7 Assay standardization

Assay heterogeneity is a practical barrier to translation (**Table 5**). The suPARnostic ELISA captures domain I and detects domain II, measuring both intact suPAR and cleaved fragments; the R&D Systems ELISA uses a polyclonal antibody mixture with different affinity for intact versus cleaved forms. The distinction matters biologically: intact suPAR binds urokinase-type plasminogen activator and vitronectin and participates in matrix remodeling, whereas the DII–DIII fragment is chemotactic via formyl peptide receptor-like 1/2 (FPRL1/2). Aptamer- and proximity extension–based proteomics platforms correlate only modestly with ELISA (r = 0.285–0.586) and show different discrimination performance (36). Preanalytical factors add further variation: serum values exceed paired plasma values, and storage conditions and freeze–thaw cycles affect stability. A turbidimetric assay on Roche Cobas platforms correlated strongly with ELISA in single-center validation (r > 0.95) (37) but has not undergone external validation against CLSI EP15-A3 or ISO 15189 standards.

**Table 5:** Assay characteristics.

Platform	Manufacturer	Detection principle	suPAR I–III	suPAR II–III	Range (ng/mL)	FSGS cut-off (ng/mL) (26)
suPARnostic ELISA	ViroGates, Denmark	Sandwich ELISA (anti-DI capture, anti-DII detection)	Yes	Yes	0.1–22.0	4.644
R&D Systems Quantikine	R&D Systems, USA	Sandwich ELISA (polyclonal mixture)	Yes	Variable	0.062–4.0	2.789
SOMAscan	SomaLogic, USA	Aptamer-based proteomics	Variable	Variable	N/A	Not established
Olink	Olink Proteomics, Sweden	Proximity extension assay	Variable	Variable	N/A	Not established
Roche Cobas turbidimetric	Roche Diagnostics	Turbidimetric immunoassay	Yes	Yes	0.5–20.0	Not established

The clinical consequence is concrete: a sample reading 3.5 ng/mL falls below the proposed FSGS cut-off on one platform and above it on the other (26). This comparison is a strictly analytical illustration of platform discordance, not an endorsement of either cut-off for clinical decision-making; both thresholds were derived from a single FSGS cohort and lack external validation in incident CKD or cardiovascular disease populations. Without internationally accepted reference materials and platform-specific calibration, absolute suPAR values cannot be compared across laboratories, and universal risk thresholds remain out of reach.

### 3.8 Certainty of evidence

**Table 4** presents the GRADE summary; domain-level ratings are shown in **Supplementary Figures S5** and **S6**. Certainty was moderate for incident CKD in preserved kidney function; low for progression of established CKD, cardiovascular events and mortality, heart failure outcomes, and microvascular complications in diabetes; and very low for pediatric CKD progression, FSGS diagnosis and prognosis, and incident type 2 diabetes.

## 4 Discussion

### 4.1 Principal findings

Across 20 studies and 31,416 participants, suPAR behaved as a consistent but non-specific marker of innate immune activation. In adults with preserved kidney function it predicted incident CKD and eGFR decline, yet the improvement in discrimination was small (absolute C-statistic increases of **0.027–0.08**). It did not diagnose FSGS, and prognostic evidence in FSGS hinged on one moderate-quality study. In pediatric CKD and in metabolic cohorts, the association vanished once baseline kidney function or inflammatory markers were taken into account. Cardiovascular associations were directionally consistent but rested on observational data. Two gaps ran through the entire literature: no study stratified by sex, and none used decision curve analysis (DCA) to test clinical net benefit.

The evidence should be read at three distinct levels. Association—whether suPAR correlates with outcome—is supported by numerous observational studies. Prediction—whether suPAR adds discriminatory value beyond established markers—is supported by fewer studies reporting modest C-statistic gains and NRI. Clinical utility—whether measuring suPAR improves patient outcomes—requires demonstration of net benefit at clinically relevant thresholds using DCA, and no included study performed this analysis. The central gap in this literature is therefore the distance between association and prediction, on the one hand, and demonstrated clinical utility, on the other.

### 4.2 Biological plausibility and mechanisms

Consistent with its role as a biomarker of systemic chronic inflammation (38), the biology of suPAR fits these observations (**Figure 1**). Activated monocytes, macrophages, and neutrophils shed uPAR through GPI-specific phospholipases and proteases such as cathepsin G, and the resulting fragments act differently. Intact suPAR engages αvβ3 integrins on podocytes, and APOL1 high-risk variants bind suPAR-activated αvβ3 with higher affinity than the reference protein, offering a molecular rationale for the excess CKD risk in individuals of African ancestry (16). The DII–DIII fragment recruits further monocytes through FPRL1/2 on vascular endothelial and smooth muscle cells, feeding plaque inflammation. This dual action helps explain why suPAR retains prognostic value in atherosclerotic disease, where local myeloid inflammation drives events, even when its association with incident diabetes is attenuated by adjustment for systemic inflammation (35).

suPAR may also intersect with trained immunity, the long-term functional reprogramming of innate immune cells through metabolic and epigenetic rewiring (39, 40). Monocytes and macrophages are both the main source of circulating suPAR and central effectors of trained immunity. suPAR could therefore serve as a readout of sustained myeloid activation, and its chemotactic fragments could in principle help perpetuate that state. Links to NLRP3 inflammasome activation and IL-1β/IL-18 release (41), and to cGAS–STING signaling (42), are biologically plausible but rest on co-occurrence rather than direct evidence and should be regarded as hypotheses to be tested.

### 4.3 Interpretation of null findings

The two null studies are as informative as the positive ones. When baseline eGFR and proteinuria were fully adjusted in pediatric CKD (22), or C-reactive protein and interleukin-6 in metabolic risk (35), suPAR lost independent association. This pattern suggests that part of suPAR’s apparent prognostic weight is model-dependent and reflects renal clearance and systemic inflammation rather than organ-specific injury. Mendelian randomization could help settle causality, but no such study of suPAR and kidney outcomes was identified. Equally, the near-uniform positivity of the published literature should be interpreted with caution: biomarker studies are prone to selective reporting, and our restriction to English-language sources may have reinforced that skew.

### 4.4 Sex differences and the limits of aggregate data

Sex adds a further complication. Women have higher baseline suPAR than men, a difference partly explained by visceral adiposity (43), and sex-specific reference intervals have been published (44). In coronary artery disease cohorts, suPAR retained prognostic value in both sexes once sex-specific thresholds were applied (43, 44), whereas a heart failure with preserved ejection fraction analysis suggested prediction in men but not women (43). Applying uniform cut-offs therefore risks systematically classifying more women as high risk than biology justifies. All 20 included studies adjusted for sex as a covariate, but adjustment does not reveal whether the suPAR–outcome association itself differs by sex.

Our *post-hoc* analysis, applying published sex-specific cut-offs (44) to study-level medians, suggested that 12–28% of participants could shift risk category in 6 of 10 evaluable studies (**Supplementary Table S3**). Because this analysis used aggregate cohort medians rather than individual participant data (IPD), it is strictly hypothesis-generating and cannot establish the direction or magnitude of misclassification at the individual level; IPD meta-analyses are needed to quantify sex-specific suPAR associations and reclassification effects reliably.

### 4.5 Therapeutic implications and the WAL0921 trial

Therapeutic development is moving faster than biomarker validation. WAL0921, a humanized monoclonal antibody that neutralizes circulating suPAR, is in phase 2 testing for proteinuric glomerular disease (NCT06466135), with phase 1 data showing dose-dependent reduction of free suPAR. In that setting suPAR acts as an enrichment marker that selects patients for an anti-suPAR drug, which is conceptually different from measuring suPAR to guide routine decisions. A positive WAL0921 trial would strengthen the causal case for suPAR, but would not by itself validate suPAR measurement for risk stratification unless a companion diagnostic strategy is tested alongside.

### 4.6 Clinical translation pathway

The research agenda follows from these gaps. Assay harmonization, including sex-specific reference intervals, is the prerequisite for everything else. Prospective cohorts should then test incremental value with DCA at treatment-relevant thresholds, for example 5–20% for 5-year CKD incidence and 10–30% for major adverse cardiovascular events. Only if net benefit is demonstrated do cost-effectiveness analyses, Mendelian randomization, and ultimately randomized trials of suPAR-guided management become meaningful. The ongoing WAL0921 trial will answer the therapeutic question, not the measurement question.

### 4.7 Limitations

This review has limitations. Nineteen of 20 studies were observational, so residual confounding cannot be excluded and causality remains open. We searched English-language sources only and did not include Chinese-language databases, which may have biased the synthesis toward positive findings and limits generalizability to East Asian populations; the C-STRIDE study (10) offers partial reassurance but cannot close that gap. Assay platforms, sample types, and outcome definitions varied enough to preclude meta-analysis and to restrict comparison of absolute values. Sex-disaggregated data could not be obtained despite direct author contact. No study performed DCA, so clinical net benefit is unknown. Our quality assessment has limitations. While the Newcastle-Ottawa Scale is widely used in biomarker research, it may underestimate residual confounding and exposure measurement error. We retained NOS to maintain consistency with prior suPAR literature, but we explicitly acknowledge this as a methodological limitation. Future updates should consider employing ROBINS-E or similar domain-based tools specifically designed for observational biomarker studies. Finally, database searching began before PROSPERO registration; although screening and analysis post-dated registration and the analysis plan was not altered in light of results, we cannot exclude an increased risk of selective reporting.

## 5 Conclusions

Current evidence does not support routine clinical measurement of suPAR for diagnosis, risk stratification, or treatment guidance. suPAR consistently predicts adverse outcomes in adults with preserved kidney function and in cardiovascular disease, but incremental discrimination is modest (absolute C-statistic gain 0.027–0.08), no study has demonstrated clinical net benefit, causality is unresolved, and assays are not standardized. It has no role in diagnosing FSGS, prognostic evidence in FSGS requires replication, and in children and inflammation-adjusted metabolic cohorts it reflects rather than predicts risk. Assay harmonization with sex-specific reference intervals, sex-disaggregated analyses, prospective validation with decision curve analysis, and randomized trials of suPAR-targeted therapy are the steps required before clinical translation can be reconsidered.

## Data Availability

The original contributions presented in the study are included in the article/**Supplementary Material**. Further inquiries can be directed to the corresponding author.

## Supplementary material

Supplementary Figure 1 Forest plot of multivariable-adjusted effect estimates, CKD domain.

Supplementary Figure 2 Forest plot of multivariable-adjusted effect estimates, FSGS domain.

Supplementary Figure 3 Forest plot of multivariable-adjusted effect estimates, cardiovascular domain.

Supplementary Figure 4 Forest plot of multivariable-adjusted effect estimates, metabolic domain.

Supplementary Figure 5 GRADE evidence profile, kidney outcomes.

Supplementary Figure 6 GRADE evidence profile, cardiovascular and metabolic outcomes.

Supplementary Material 1 Complete search strategies for all databases and registries.

Supplementary Material 2 PRISMA 2020 flow diagram and list of excluded full-text studies with reasons.

Supplementary Material 3 Detailed risk-of-bias assessments (NOS, JBI, RoB 2, QUADAS-2) and GRADE evidence profiles.

Supplementary Material 4 Data extraction form template and author contact log.

Supplementary Material 5 Leave-one-out sensitivity analyses.

Supplementary Table 1 Multivariable-adjusted effect estimates for suPAR in CKD populations, including adjustment covariates.

Supplementary Table 2 Multivariable-adjusted effect estimates for suPAR in FSGS, cardiovascular, and metabolic cohorts, including adjustment covariates.

Supplementary Table 3 *Post-hoc* reclassification analysis applying sex-specific suPAR cut-offs to included studies.
