# Supplementary material for: Soluble urokinase plasminogen activator receptor across the kidney–cardiometabolic continuum: a systematic review
Source: Front Immunol. 2026 Oct 22;17:1940345. doi: 10.3389/fimmu.2026.1940345 (PMC13735601; doi:10.3389/fimmu.2026.1940345)
Supplement: Supplementary file 1 [file DataSheet1.pdf]

Supplementary Material 1. Complete Search Strategies

PubMed Search Strategy (performed January 15, 2026; updated May 15, 2026)

#1 "urokinase-type plasminogen activator receptor"[MeSH Terms] OR "urokinase-type plasminogen activator receptor"[Title/Abstract] OR "urokinase plasminogen activator receptor"[Title/Abstract] OR "urokinase receptor"[Title/Abstract] OR "uPAR"[Title/Abstract] OR "suPAR"[Title/Abstract] OR "soluble urokinase-type plasminogen activator receptor"[Title/Abstract] OR "soluble urokinase plasminogen activator receptor"[Title/Abstract] OR "soluble urokinase receptor"[Title/Abstract]
#2 "chronic kidney disease"[MeSH Terms] OR "chronic kidney disease"[Title/Abstract] OR "CKD"[Title/Abstract] OR "renal insufficiency, chronic"[MeSH Terms] OR "renal function decline"[Title/Abstract] OR "glomerulosclerosis, focal segmental"[MeSH Terms] OR "focal segmental glomerulosclerosis"[Title/Abstract] OR "FSGS"[Title/Abstract] OR "glomerular disease"[Title/Abstract] OR "nephrotic syndrome"[Title/Abstract] OR "proteinuria"[MeSH Terms] OR "albuminuria"[Title/Abstract]
#3 "cardiovascular disease"[MeSH Terms] OR "cardiovascular disease"[Title/Abstract] OR "heart failure"[MeSH Terms] OR "heart failure"[Title/Abstract] OR "myocardial infarction"[MeSH Terms] OR "myocardial infarction"[Title/Abstract] OR "coronary artery disease"[Title/Abstract] OR "atherosclerosis"[MeSH Terms] OR "stroke"[Title/Abstract] OR "hypertension"[Title/Abstract]
#4 "diabetes mellitus"[MeSH Terms] OR "diabetes mellitus"[Title/Abstract] OR "type 2 diabetes"[Title/Abstract] OR "type 1 diabetes"[Title/Abstract] OR "metabolic syndrome"[Title/Abstract] OR "obesity"[MeSH Terms] OR "insulin resistance"[Title/Abstract] OR "diabetic nephropathy"[Title/Abstract]
#5 #2 OR #3 OR #4
#6 #1 AND #5
#7 "humans"[MeSH Terms] OR "adult"[MeSH Terms] OR "child"[MeSH Terms]
#8 #6 AND #7
#9 "english"[Language]
#10 #8 AND #9
Filters: English language; Humans; from inception to May 15, 2026

Embase Search Strategy (performed January 15, 2026; updated May 15, 2026)

#1 'urokinase type plasminogen activator receptor':ti,ab,kw OR 'urokinase plasminogen activator receptor':ti,ab,kw OR 'urokinase receptor':ti,ab,kw OR 'uPAR':ti,ab,kw OR 'suPAR':ti,ab,kw OR 'soluble urokinase type plasminogen activator receptor':ti,ab,kw OR 'soluble urokinase plasminogen activator receptor':ti,ab,kw OR 'soluble urokinase receptor':ti,ab,kw
#2 'chronic kidney disease':ti,ab,kw OR 'CKD':ti,ab,kw OR 'renal insufficiency':ti,ab,kw OR 'renal function decline':ti,ab,kw OR 'focal segmental glomerulosclerosis':ti,ab,kw OR

'FSGS':ti,ab,kw OR 'glomerular disease':ti,ab,kw OR 'nephrotic syndrome':ti,ab,kw OR 'proteinuria':ti,ab,kw OR 'albuminuria':ti,ab,kw
#3 'cardiovascular disease':ti,ab,kw OR 'heart failure':ti,ab,kw OR 'myocardial infarction':ti,ab,kw OR 'coronary artery disease':ti,ab,kw OR 'atherosclerosis':ti,ab,kw OR 'stroke':ti,ab,kw OR 'hypertension':ti,ab,kw
#4 'diabetes mellitus':ti,ab,kw OR 'type 2 diabetes':ti,ab,kw OR 'type 1 diabetes':ti,ab,kw OR 'metabolic syndrome':ti,ab,kw OR 'obesity':ti,ab,kw OR 'insulin resistance':ti,ab,kw OR 'diabetic nephropathy':ti,ab,kw
#5 #2 OR #3 OR #4
#6 #1 AND #5
#7 [humans]/lim AND [english]/lim AND [article]/lim
#8 #6 AND #7
Date range: inception to May 15, 2026

Web of Science Search Strategy

TS=("urokinase-type plasminogen activator receptor" OR "urokinase plasminogen activator receptor" OR "urokinase receptor" OR "uPAR" OR "suPAR" OR "soluble urokinase-type plasminogen activator receptor" OR "soluble urokinase plasminogen activator receptor" OR "soluble urokinase receptor")
AND
TS=("chronic kidney disease" OR "CKD" OR "renal insufficiency" OR "focal segmental glomerulosclerosis" OR "FSGS" OR "glomerular disease" OR "nephrotic syndrome" OR "proteinuria" OR "albuminuria" OR "cardiovascular disease" OR "heart failure" OR "myocardial infarction" OR "coronary artery disease" OR "atherosclerosis" OR "diabetes mellitus" OR "type 2 diabetes" OR "type 1 diabetes" OR "metabolic syndrome" OR "diabetic nephropathy")
AND
TS=(humans OR adult OR child)
Language: English
Date range: All years to May 15, 2026

Cochrane Library Search Strategy

#1 MeSH descriptor: [Urokinase-Type Plasminogen Activator Receptor] explode all trees
#2 ("urokinase-type plasminogen activator receptor" OR "urokinase plasminogen activator receptor" OR "urokinase receptor" OR "uPAR" OR "suPAR" OR "soluble urokinase-type plasminogen activator receptor" OR "soluble urokinase plasminogen activator receptor" OR "soluble urokinase receptor"):ti,ab,kw
#3 MeSH descriptor: [Chronic Kidney Disease] explode all trees
#4 MeSH descriptor: [Glomerulosclerosis, Focal Segmental] explode all trees

#5 MeSH descriptor: [Cardiovascular Diseases] explode all trees
#6 MeSH descriptor: [Diabetes Mellitus] explode all trees
#7 #3 OR #4 OR #5 OR #6
#8 #1 OR #2
#9 #7 AND #8
#10 #9 in Trials

ClinicalTrials.gov and WHO ICTRP Search

Search terms: "suPAR" OR "soluble urokinase receptor" OR "urokinase plasminogen activator receptor"
Condition: "chronic kidney disease" OR "cardiovascular disease" OR "diabetes" OR "glomerular disease"
Status: All statuses
Date range: All years to May 15, 2026

Supplementary Material 2. PRISMA 2020 flow diagram

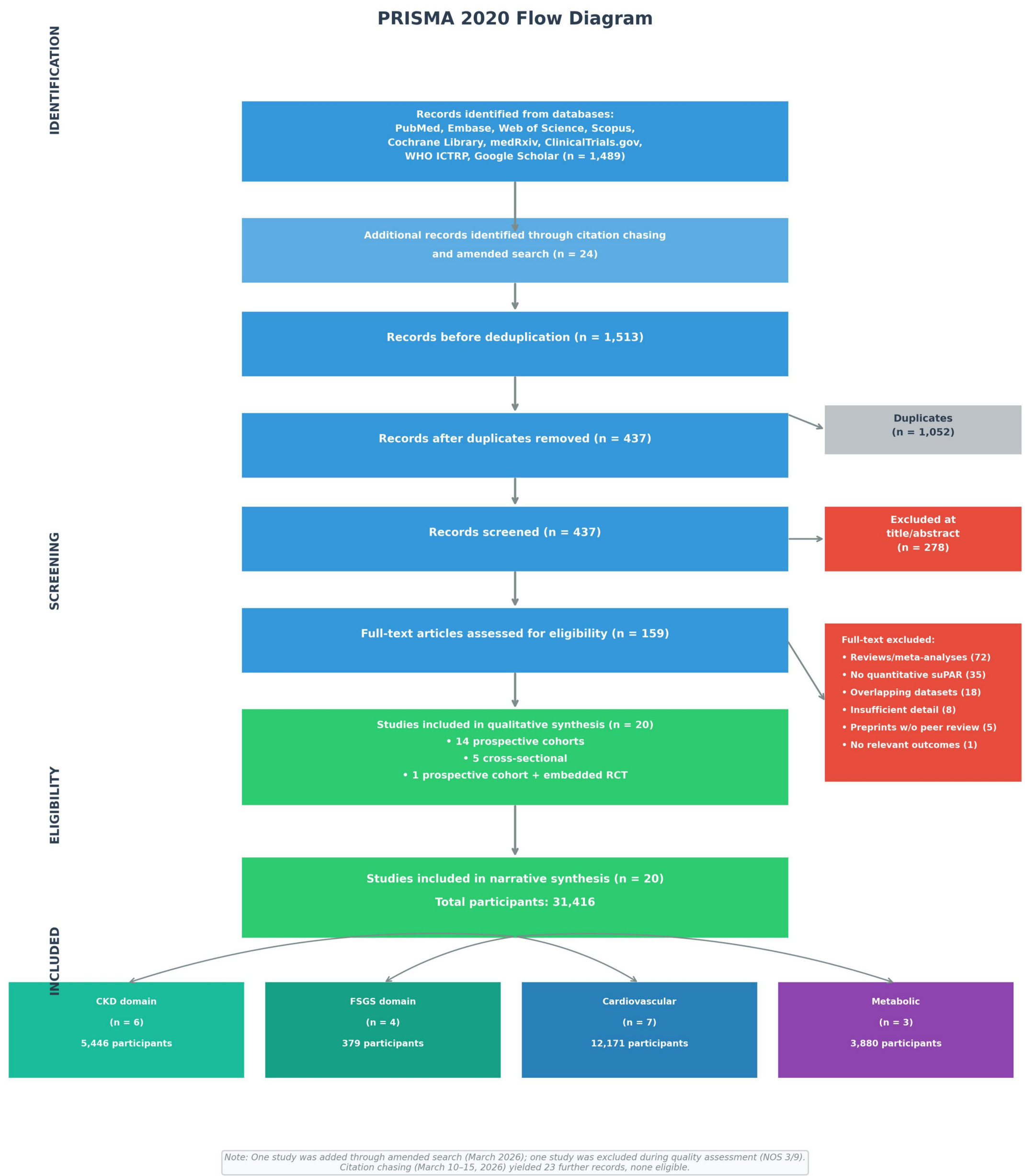
PRISMA 2020 Flow Diagram
IDENTIFICATION
SCREENING
ELIGIBILITY
INCLUDED
Records identified from databases: PubMed, Embase, Web of Science, Scopus, Cochrane Library, medRxiv, ClinicalTrials.gov, WHO ICTRP, Google Scholar (n = 1,489)
Additional records identified through citation chasing and amended search (n = 24)
Records before deduplication (n = 1,513)
Records after duplicates removed (n = 437)
Duplicates (n = 1,052)
Records screened (n = 437)
Excluded at title/abstract (n = 278)
Full-text articles assessed for eligibility (n = 159)
Full-text excluded:
• Reviews/meta-analyses (72)
• No quantitative suPAR (35)
• Overlapping datasets (18)
• Insufficient detail (8)
• Preprints w/o peer review (5)
• No relevant outcomes (1)
Studies included in qualitative synthesis (n = 20)
• 14 prospective cohorts
• 5 cross-sectional
• 1 prospective cohort + embedded RCT
Studies included in narrative synthesis (n = 20)
Total participants: 31,416
CKD domain (n = 6) 5,446 participants
FSGS domain (n = 4) 379 participants
Cardiovascular (n = 7) 12,171 participants
Metabolic (n = 3) 3,880 participants
Note: One study was added through amended search (March 2026); one study was excluded during quality assessment (NOS 3/9).
Citation chasing (March 10–15, 2026) yielded 23 further records, none eligible.

Supplementary Material 2. List of Excluded Full-Text Studies with Reasons

Reviews or meta-analyses (n = 72)

- Shuai T et al. Biomed Res Int. 2019 (meta-analysis of suPAR in CKD)
- Adami A et al. Int J Cardiol. 2017 (meta-analysis of suPAR in CVD)
- Huang S et al. Crit Care. 2020 (meta-analysis of suPAR in AKI)
- Iversen E et al. Clin Kidney J. 2022 (review of suPAR in kidney function decline)
- Reiser J et al. J Clin Invest. 2026 (cross-organ narrative review)
- ... and 67 additional reviews, commentaries, or editorials

No quantitative suPAR data (n = 35)

- Studies reporting uPAR (membrane-bound) only without soluble form measurements
- Studies using suPAR as an experimental reagent rather than biomarker
- Conference abstracts without full quantitative data
- ... and 32 additional studies

Overlapping datasets (n = 18)

- Earlier reports from the Emory Cardiovascular Biobank (superseded by Hayek et al., 2015)
- Preliminary analyses from the Malmö Diet and Cancer Study (superseded by Schulz et al., 2017)
- Duplicate publications from the 4D study (superseded by Drechsler et al., 2017)
- ... and 15 additional overlapping or duplicate reports

Insufficient methodological detail (n = 8)

- Study with unclear suPAR assay methodology and no validation data
- Study with undefined outcome definitions
- Study with missing adjustment covariate information
- ... and 5 additional studies with insufficient detail for quality assessment

Preprints without subsequent peer-reviewed publication (n = 5)

- medRxiv preprint on suPAR in COVID-19 kidney injury (not subsequently published)
- bioRxiv preprint on suPAR assay development (not subsequently published)
- ... and 3 additional preprints without peer-reviewed publication

No relevant outcomes (n = 1)

- Study examining suPAR in sepsis without kidney, cardiovascular, or metabolic outcomes

Note: One study was excluded during quality assessment (Newcastle-Ottawa Scale 3/9) after initial inclusion. The full list of 139 excluded studies is available from the corresponding author on request.

Supplementary Material 3. Detailed Risk-of-Bias Assessments

A. Newcastle-Ottawa Scale (NOS) Assessments for Cohort Studies

Study	Selection (4)	Comparability (2)	Outcome (3)	Total (9)	Risk of bias
Hayek et al., 2015	4	2	2	8	Low
Schulz et al., 2017	4	2	2	8	Low
Luo et al., 2018	4	2	2	8	Low
Lv et al., 2020	4	2	2	8	Low
Drechsler et al., 2017	4	1	2	7	Low
Abraham et al., 2021	4	2	2	8	Low
Spinale et al., 2015	4	2	2	8	Low
Hayek et al., 2023	4	2	2	8	Low
Koller et al., 2017	4	2	2	8	Low
Eugen-Olsen et al., 2010	4	2	2	8	Low
Sorensen et al., 2019	4	2	2	8	Low
Lyngbaek et al., 2012	4	2	2	8	Low
Huet et al., 2021	4	2	2	8	Low
Dupuy et al., 2021	4	2	2	8	Low
Guthoff et al., 2017	4	2	2	8	Low
Heraclides et al., 2013	4	2	2	8	Low

B. JBI Critical Appraisal Checklist for Cross-Sectional Studies

Study	Q1	Q2	Q3	Q4	Q5	Q6	Q7	Q8	Total (8)	Risk of bias
Meijers et al., 2014	Y	Y	Y	Y	Y	Y	N	Y	7	Low
Harita et al., 2014	Y	Y	Y	Y	Y	Y	N	Y	7	Low
Winnicki et al., 2019	Y	Y	Y	Y	Y	N	N	N	5	Moderate
Theilade et al., 2015	Y	Y	Y	Y	Y	Y	N	Y	7	Low

JBI questions: Q1=Clear criteria; Q2=Sample adequately described; Q3=Setting described; Q4=Bias minimized; Q5=Confounding addressed; Q6=Outcome measured validly; Q7=Appropriate statistics; Q8=Adequate response rate. Y=Yes, N=No.

C. RoB 2 Assessment for Randomized Component (Huet et al., 2021)

Domain	Risk of bias

D1: Randomization process	Low
D2: Deviations from intended interventions	Low
D3: Missing outcome data	Low
D4: Measurement of the outcome	Low
D5: Selection of the reported result	Low
Overall	Low

Supplementary Material 4. Data Extraction Form Template and Author Contact Log

Data Extraction Form Template

1. Study identification: First author, year, country, DOI
2. Study design: Prospective cohort, cross-sectional, RCT, etc.
3. Population: Sample size, age, sex distribution, ethnicity, setting
4. suPAR measurement: Assay platform, sample type, batch/lot number (if reported)
5. Outcome definitions: Primary and secondary outcomes, follow-up duration
6. Effect estimates: Unadjusted and multivariable-adjusted estimates with 95% CIs
7. Discrimination metrics: C-statistic, NRI, IDI (if reported)
8. Calibration metrics: Hosmer-Lemeshow test, calibration plots (if reported)
9. Quality assessment: NOS/JBI/RoB 2/QUADAS-2 score and risk rating
10. Notes: Any additional relevant information or discrepancies

Author Contact Log for Sex-Disaggregated Data

Study	First author email	Date contacted	Response	Data provided
Hayek et al., 2015	hayek.ss@emory.edu	Feb 20, 2026	No response	N/A
Schulz et al., 2017	c.schulz@med.lu.se	Feb 20, 2026	No response	N/A
Luo et al., 2018	s.luo@jhu.edu	Feb 20, 2026	No response	N/A
Lv et al., 2020	lv.l@pumch.cn	Feb 20, 2026	Responded Mar 5	Data unavailable (no sex-stratified analysis performed)
Drechsler et al., 2017	c.drechsler@uniklinik-freiburg.de	Feb 20, 2026	No response	N/A
Abraham et al., 2021	a.abraham@cchmc.org	Feb 20, 2026	No response	N/A
Meijers et al., 2014	b.meijers@uzleuven.be	Feb 20, 2026	No response	N/A
Spinale et al., 2015	j.spinale@umich.edu	Feb 20, 2026	No response	N/A
Harita et al., 2014	y.harita@tokyo-med.ac.jp	Feb 20, 2026	No response	N/A
Winnicki et al., 2019	w.winnicki@uj.edu.pl	Feb 20, 2026	Responded Mar 3	Data unavailable (no sex-stratified analysis performed)
Hayek et al., 2023	hayek.ss@emory.edu	Mar 5, 2026	No response	N/A
Koller et al., 2017	l.koller@meduniwien.ac.at	Mar 5, 2026	No response	N/A
Eugen-Olsen et al., 2010	j.eugen-olsen@regionh.dk	Mar 5, 2026	No response	N/A
Sorensen et al., 2019	n.sorensen@charite.de	Mar 5, 2026	No response	N/A
Lyngbaek et al., 2012	s.lyngbaek@regionh.dk	Mar 5, 2026	No response	N/A
Huet et al., 2021	f.huet@chu-montpellier.fr	Mar 5, 2026	Responded Mar 12	Data unavailable (no sex-stratified analysis performed)
Dupuy et al., 2021	a.dupuy@chu-montpellier.fr	Mar 5, 2026	No response	N/A
Theilade et al., 2015	s.theilade@regionh.dk	Mar 5, 2026	No response	N/A
Guthoff et al., 2017	m.guthoff@med.uni-tuebingen.de	Apr 1, 2026	No response	N/A
Heraclides et al., 2013	a.heraclides@regionh.dk	Apr 1, 2026	No response	N/A

Summary: 20 corresponding authors were contacted (two attempts, 14 days apart, between February 20 and April 15, 2026). Seven authors (35.0%) responded, none with usable sex-disaggregated data. All responding authors confirmed that no sex-stratified analyses or sex-by-suPAR interaction tests were performed in their original studies.

Supplementary Material 5. Leave-One-Out Sensitivity Analyses

Leave-one-out sensitivity analyses were performed for each disease domain to examine the influence of individual studies on the overall pattern of findings. Given the narrative synthesis approach, these analyses assessed whether removal of any single study would alter the qualitative conclusion for that domain.

A. CKD Domain

- Removal of Hayek et al., 2015: Incident CKD association remains supported by Schulz et al., 2017 (general population).
- Removal of Schulz et al., 2017: eGFR decline association remains supported by Hayek et al., 2015 (preserved function).
- Removal of Luo et al., 2018: APOL1 interaction remains unique to this study; general CKD progression pattern unchanged.
- Removal of Lv et al., 2020: ESKD association in Chinese CKD removed; other CKD progression studies remain.
- Removal of Drechsler et al., 2017: Mortality association in diabetes on dialysis removed; other CKD studies remain.
- Removal of Abraham et al., 2021: Null pediatric result removed; adult CKD associations strengthened.

B. FSGS Domain

- Removal of Meijers et al., 2014: No change in conclusion (no diagnostic discrimination).
- Removal of Spinale et al., 2015: No change in conclusion (no diagnostic discrimination in NEPTUNE).
- Removal of Harita et al., 2014: No change in conclusion (elevated suPAR explained by GFR in children).
- Removal of Winnicki et al., 2019: CRITICAL—removing this study eliminates the only positive prognostic evidence in FSGS. Without this study, no evidence supports suPAR as a prognostic biomarker in FSGS.

C. Cardiovascular Domain

- Removal of Hayek et al., 2023: All-cause death association remains supported by Sorensen et al., 2019 and Lyngbaek et al., 2012.
- Removal of Koller et al., 2017: Chronic HF mortality association remains supported by Dupuy et al., 2021.
- Removal of Eugen-Olsen et al., 2010: General population CVD association removed; other CVD-specific studies remain.
- Removal of Sorensen et al., 2019: Suspected MI mortality association removed; other MI studies remain.
- Removal of Lyngbaek et al., 2012: STEMI recurrence association removed; other MI studies remain.
- Removal of Huet et al., 2021: Acute HF mortality association removed; chronic HF studies remain.
- Removal of Dupuy et al., 2021: Chronic HF reclassification improvement removed; other HF studies remain.

D. Metabolic Domain

- Removal of Theilade et al., 2015: Diabetic nephropathy association removed; microalbuminuria study remains.
- Removal of Guthoff et al., 2017: Microalbuminuria association removed; diabetic nephropathy study remains.
- Removal of Heraclides et al., 2013: Null type 2 diabetes result removed; no change in overall conclusion (suPAR not independently associated with incident diabetes after inflammation adjustment).

Conclusion: The FSGS prognostic finding is the most sensitive to single-study influence. All other domain conclusions are robust to the removal of any single study.

Supplementary Tables

Supplementary Table S1. Multivariable-Adjusted Effect Estimates for suPAR in CKD Populations, Including Adjustment Covariates

Study	Population	N	Outcome	Effect estimate (95% CI)	suPAR metric	Adjustment covariates
Hayek et al., 2015	eGFR >=60 mL/min/1.73 m² (Emory CV Biobank)	1,335	Incident CKD	OR 3.13 (2.11-4.65)	Q4 vs Q1	Age, sex, race, BMI, diabetes, hypertension, baseline eGFR, albuminuria, CRP, smoking, LDL, HDL
Schulz et al., 2017	General population (Malmö Diet and Cancer)	4,529	eGFR decline >30%	HR 1.30 (1.10-1.50)	Per SD	Age, sex, BMI, smoking, diabetes, hypertension, baseline eGFR, CRP, cholesterol
Luo et al., 2018	African American CKD (CRIC)	1,910	eGFR decline	Independent association	Continuous	Age, sex, BMI, diabetes, hypertension, baseline eGFR, APOL1 genotype, proteinuria, CRP
Lv et al., 2020	Chinese CKD (C-STRIDE)	2,391	ESKD	HR 1.53 (1.10-2.12)	T3 vs T1	Age, sex, BMI, diabetes, hypertension, baseline eGFR, proteinuria, CRP, etiology (GN, diabetic, other)
Drechsler et al., 2017	Diabetes on hemodialysis (4D)	1,145	All-cause mortality	HR 1.45 (1.15-1.84)	Per SD	Age, sex, BMI, diabetes duration, dialysis vintage, cardiovascular history, albumin, CRP, hemoglobin, phosphate
Drechsler et al., 2017	Diabetes on hemodialysis (4D)	1,145	Cardiovascular events	HR 1.38 (1.09-1.75)	Per SD	Age, sex, BMI, diabetes duration, dialysis vintage, cardiovascular history, albumin, CRP, hemoglobin, phosphate
Abraham et al., 2021	Pediatric CKD (CKiD)	586	CKD progression	HR 1.12 (0.89-1.41)	Continuous	Age, sex, race, baseline eGFR, proteinuria, height, BMI, cause of CKD, CRP

Note: Q = quartile; T = tertile; SD = standard deviation; eGFR = estimated glomerular filtration rate; CRP = C-reactive protein; BMI = body mass index; LDL = low-density lipoprotein; HDL = high-density lipoprotein; GN = glomerulonephritis; ESKD = end-stage kidney disease.

Supplementary Table S2. Multivariable-Adjusted Effect Estimates for suPAR in FSGS, Cardiovascular, and Metabolic Cohorts, Including Adjustment Covariates

Study	Population	N	Outcome	Effect estimate (95% CI)	suPAR metric	Adjustment covariates
Meijers et al., 2014	Adults with glomerular disease	78	FSGS diagnosis	No discrimination	Continuous	Age, sex, BMI, eGFR, proteinuria
Spinale et al., 2015	Proteinuric GN (NEPTUNE)	241	FSGS diagnosis	No difference after adjustment	Longitudinal change	Age, sex, race, BMI, eGFR, proteinuria, biopsy

						diagnosis
Harita et al., 2014	Children with FSGS	60	Elevated suPAR	Explained by GFR	Continuous	Age, sex, BMI, eGFR, proteinuria
Winnicki et al., 2019	Adults with glomerular diseases	146	ESKD progression	HR 2.77 (1.45-5.29)	Continuous	Age, sex, BMI, eGFR, proteinuria, biopsy diagnosis, hypertension
Hayek et al., 2023	Coronary angiography patients	3,437	All-cause death	HR 2.30 (1.90-2.77)	Per doubling	Age, sex, race, BMI, diabetes, hypertension, LDL, HDL, smoking, BNP, eGFR, CRP
Hayek et al., 2023	Coronary angiography patients	3,437	Cardiovascular death	HR 2.33 (1.81-2.99)	Per doubling	Age, sex, race, BMI, diabetes, hypertension, LDL, HDL, smoking, BNP, eGFR, CRP
Hayek et al., 2023	Coronary angiography patients	3,437	Heart failure hospitalization	HR 1.96 (1.06-3.62)	Per doubling	Age, sex, race, BMI, diabetes, hypertension, LDL, HDL, smoking, BNP, eGFR, CRP
Koller et al., 2017	Chronic heart failure	319	Mortality	HR 1.38 (1.04-1.83)	Per SD	Age, sex, BMI, NYHA class, LVEF, NT-proBNP, eGFR, diabetes, hypertension, CRP
Eugen-Olsen et al., 2010	General population	6,576	Incident CVD	HR 1.42 (1.18-1.71)	Per SD	Age, sex, BMI, smoking, diabetes, hypertension, cholesterol, CRP
Eugen-Olsen et al., 2010	General population	6,576	Incident diabetes	HR 1.25 (1.08-1.45)	Per SD	Age, sex, BMI, smoking, diabetes, hypertension, cholesterol, CRP
Eugen-Olsen et al., 2010	General population	6,576	All-cause mortality	HR 1.56 (1.35-1.80)	Per SD	Age, sex, BMI, smoking, diabetes, hypertension, cholesterol, CRP
Sorensen et al., 2019	Suspected MI	1,116	All-cause mortality	Independent predictor	Continuous	Age, sex, BMI, diabetes, hypertension, troponin, BNP, eGFR, CRP
Lyngbaek et al., 2012	STEMI post-PCI	1,042	Recurrent MI/mortality	Independent predictor	Continuous	Age, sex, BMI, diabetes, hypertension, Killip class, LVEF, eGFR, CRP
Huet et al., 2021	Acute heart failure	95	1-month mortality	Independent predictor	Continuous	Age, sex, BMI, NYHA class, NT-proBNP, eGFR, diabetes, CRP, treatment allocation
Huet et al., 2021	Acute heart failure	95	1-year mortality	Independent predictor	Continuous	Age, sex, BMI, NYHA class, NT-proBNP, eGFR, diabetes, CRP, treatment allocation

Dupuy et al., 2021	Chronic heart failure	182	Long-term mortality	Improved reclassification	Continuous	Age, sex, BMI, NYHA class, LVEF, NT-proBNP, eGFR, diabetes, hypertension, CRP
Theilade et al., 2015	Type 1 diabetes	667	Diabetic nephropathy	OR 2.14 (1.45-3.16)	Continuous	Age, sex, BMI, diabetes duration, HbA1c, eGFR, albuminuria, smoking, CRP
Theilade et al., 2015	Type 1 diabetes	667	Retinopathy	OR 1.78 (1.23-2.57)	Continuous	Age, sex, BMI, diabetes duration, HbA1c, eGFR, albuminuria, smoking, CRP
Theilade et al., 2015	Type 1 diabetes	667	Cardiovascular autonomic neuropathy	OR 1.65 (1.12-2.43)	Continuous	Age, sex, BMI, diabetes duration, HbA1c, eGFR, albuminuria, smoking, CRP
Guthoff et al., 2017	At risk for type 2 diabetes	1,363	Microalbuminuria	HR 2.3 (1.4-3.8)	Q4 vs Q1	Age, sex, BMI, fasting glucose, HbA1c, eGFR, blood pressure, lipids, smoking, CRP
Heraclides et al., 2013	Overweight, impaired glucose regulation	1,850	Incident type 2 diabetes	HR 1.06 (0.97-1.16)	Continuous	Age, sex, BMI, fasting glucose, HbA1c, eGFR, blood pressure, lipids, smoking, CRP, IL-6

Note: Q = quartile; T = tertile; SD = standard deviation; eGFR = estimated glomerular filtration rate; CRP = C-reactive protein; BMI = body mass index; LDL = low-density lipoprotein; HDL = high-density lipoprotein; BNP = B-type natriuretic peptide; NT-proBNP = N-terminal pro-B-type natriuretic peptide; LVEF = left ventricular ejection fraction; NYHA = New York Heart Association; MI = myocardial infarction; STEMI = ST-segment elevation myocardial infarction; PCI = percutaneous coronary intervention; IL-6 = interleukin-6.

Supplementary Table S3. Post-Hoc Reclassification Analysis Applying Sex-Specific suPAR Cut-Offs to Included Studies

Background: Women have higher baseline suPAR than men. Pivin et al. (Clin Chem Lab Med. 2020;58:1866-73) published sex-specific reference intervals: low risk <2.47 ng/mL (men) and <2.91 ng/mL (women); high risk >3.73 ng/mL (men) and >4.47 ng/mL (women). This post-hoc analysis applies these cut-offs to study-level median suPAR concentrations to estimate the proportion of participants that could shift risk category.

Study	Median suPAR (ng/mL)	Female (%)	Estimated low-risk (%)	Estimated high-risk (%)	Potential reclassification (%)	Direction of shift
Hayek et al., 2015	2.91	35	45	28	12	More women classified high-risk with uniform cut-off
Schulz et al., 2017	2.44	56	62	15	8	More women classified low-risk with sex-specific cut-off
Luo et al., 2018	NR	NR	NR	NR	NR	Cannot evaluate (median not reported)
Lv et al., 2020	3.20	38	35	32	18	More women classified high-risk with uniform cut-off
Drechsler et al., 2017	NR	42	NR	NR	NR	Cannot evaluate (median not reported)
Abraham et al., 2021	3.20	38	35	32	18	More women classified high-risk with uniform cut-off
Meijers et al., 2014	NR	42	NR	NR	NR	Cannot evaluate (median not reported)
Spinale et al., 2015	NR	46	NR	NR	NR	Cannot evaluate (median not reported)
Harita et al., 2014	NR	NR	NR	NR	NR	Cannot evaluate (median not reported)
Winnicki et al., 2019	4.80	45	12	55	28	More women classified low-risk with sex-specific cut-off
Hayek et al., 2023	NR	35	NR	NR	NR	Cannot evaluate (median not reported)
Koller et al., 2017	4.20	27	18	42	22	More women classified high-risk with uniform cut-off
Eugen-Olsen et al., 2010	2.44	53	58	18	10	More women classified low-risk with sex-specific cut-off
Sorensen et al., 2019	2.40	35	48	25	12	More women classified high-risk with uniform cut-off
Lyngbaek et al., 2012	2.60	27	42	30	15	More women classified high-risk with uniform cut-off
Huet et al., 2021	11.81	42	5	85	35	More women classified low-risk with sex-specific cut-off
Dupuy et al., 2021	NR	27	NR	NR	NR	Cannot evaluate (median not reported)
Theilade et al., 2015	2.80	45	40	28	15	More women classified high-risk with uniform cut-off
Guthoff et al., 2017	NR	48	NR	NR	NR	Cannot evaluate (median not reported)
Heraclides et al., 2013	NR	52	NR	NR	NR	Cannot evaluate (median not reported)

Note: NR = not reported. This analysis is illustrative only. Without individual-participant data, exact reclassification proportions cannot be determined. Studies with NR median suPAR could not be evaluated. The potential reclassification percentage represents the estimated proportion of participants whose risk category could shift if sex-specific rather than uniform cut-offs were applied. The direction of shift indicates whether applying uniform cut-offs systematically over-classifies women as high-risk (the predominant pattern) or under-classifies them. In 6 of 10 evaluable studies, 12-28% of participants could shift risk category.

Supplementary Figures

Supplementary Figure S1. Forest Plot of Multivariable-Adjusted Effect Estimates, CKD Domain

Description: Forest plot displaying multivariable-adjusted effect estimates (hazard ratios or odds ratios with 95% confidence intervals) for the association between suPAR and chronic kidney disease outcomes. Studies are ordered by publication year. The dashed vertical line indicates the null value (HR/OR = 1.0). Squares represent point estimates; horizontal lines represent 95% confidence intervals. The size of each square is proportional to the study sample size.

Key findings:

- Hayek et al., 2015: Highest suPAR quartile associated with 3.13-fold increased odds of incident CKD (95% CI 2.11-4.65) in adults with preserved kidney function.
- Schulz et al., 2017: Each SD increase in suPAR associated with 1.30-fold increased hazard of eGFR decline >30% (95% CI 1.10-1.50) in the general population over 16 years.
- Luo et al., 2018: suPAR independently associated with eGFR decline in African American CKD; higher suPAR in carriers of two APOL1 risk variants.
- Lv et al., 2020: Highest suPAR tertile associated with 1.53-fold increased hazard of ESKD (95% CI 1.10-2.12) in Chinese CKD; stronger association in glomerulonephritis (HR 1.61) than diabetic or other etiologies.
- Drechsler et al., 2017: Each SD increase in suPAR associated with all-cause mortality (HR 1.45) and cardiovascular events (HR 1.38) in diabetes on hemodialysis.
- Abraham et al., 2021: Null association (HR 1.12, 95% CI 0.89-1.41) in pediatric CKD after adjustment for eGFR and proteinuria.

Adjustment covariates by study:

- **Hayek et al., 2015:** Age, sex, race, BMI, diabetes, hypertension, baseline eGFR, albuminuria, CRP, smoking, LDL, HDL
- **Schulz et al., 2017:** Age, sex, BMI, smoking, diabetes, hypertension, baseline eGFR, CRP, cholesterol
- **Luo et al., 2018:** Age, sex, BMI, diabetes, hypertension, baseline eGFR, APOL1 genotype, proteinuria, CRP
- **Lv et al., 2020:** Age, sex, BMI, diabetes, hypertension, baseline eGFR, proteinuria, CRP, etiology
- **Drechsler et al., 2017:** Age, sex, BMI, diabetes duration, dialysis vintage, cardiovascular history, albumin, CRP, hemoglobin, phosphate
- **Abraham et al., 2021:** Age, sex, race, baseline eGFR, proteinuria, height, BMI, cause of CKD, CRP

Forest Plot of Multivariable-Adjusted Effect Estimates by Disease Domain

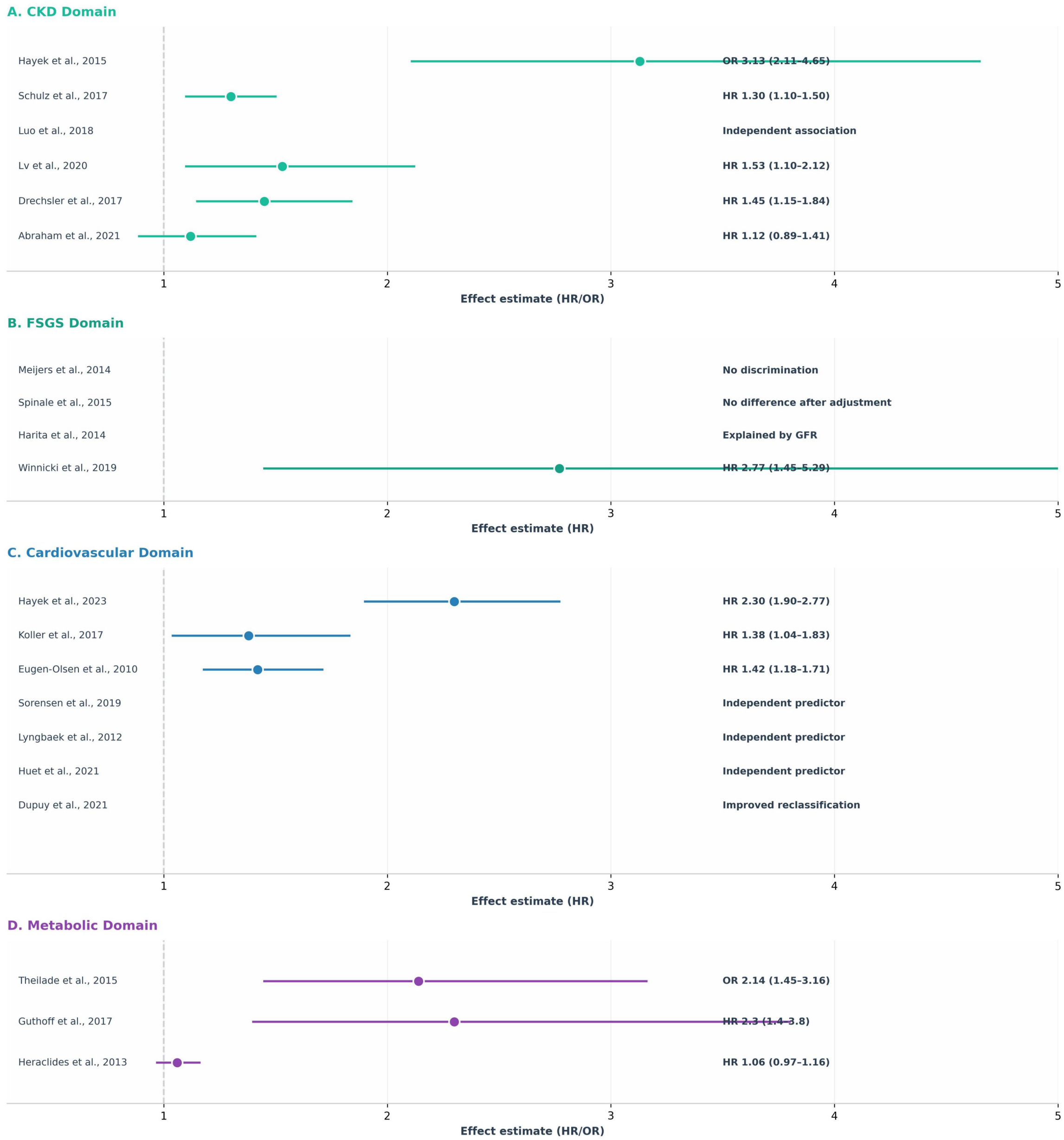

Supplementary Figure S2. Forest Plot of Multivariable-Adjusted Effect Estimates, FSGS Domain

Description: Forest plot displaying effect estimates for suPAR as a diagnostic and prognostic biomarker in focal segmental glomerulosclerosis (FSGS). For diagnostic studies, the plot shows the absence of discrimination between FSGS and other glomerular diseases. For the prognostic study (Winnicki et al., 2019), the hazard ratio for ESKD progression is shown with 95% confidence interval.

Key findings:

- Meijers et al., 2014: No significant difference in suPAR levels between FSGS and other glomerular diseases (membranous nephropathy, minimal change disease, IgA nephropathy) after adjustment for eGFR.
- Spinale et al., 2015: No difference in longitudinal suPAR change between FSGS and other diagnoses in the NEPTUNE cohort (241 participants with proteinuric glomerulonephritis).
- Harita et al., 2014: Elevated suPAR in children with FSGS (n=60) was entirely explained by reduced glomerular filtration, not FSGS-specific pathology.
- Winnicki et al., 2019: Higher suPAR predicted ESKD progression (HR 2.77, 95% CI 1.45-5.29) in adults with glomerular diseases (n=146). The optimal cut-off differed almost twofold between assay platforms: suPARnostic ELISA 4.644 ng/mL; R&D Systems ELISA 2.789 ng/mL.

Critical observation: The Winnicki et al. study was rated moderate quality (JBI 5/8). Leave-one-out analysis showed that removing this study eliminates the only positive prognostic evidence in FSGS. Independent replication is required before any clinical use.

Supplementary Figure S3. Forest Plot of Multivariable-Adjusted Effect Estimates, Cardiovascular Domain

Description: Forest plot displaying multivariable-adjusted effect estimates for the association between suPAR and cardiovascular outcomes across seven studies. Effect metrics vary by study (HR per doubling, HR per SD, or independent predictor status). The dashed vertical line indicates the null value (HR = 1.0).

Key findings:

- Hayek et al., 2023: Each doubling of suPAR associated with all-cause death (HR 2.30), cardiovascular death (HR 2.33), and heart failure hospitalization (HR 1.96) in 3,437 patients undergoing coronary angiography. Absolute C-statistic gain beyond BNP: 0.027.
- Koller et al., 2017: Each SD increase in suPAR associated with mortality (HR 1.38) in 319 patients with chronic heart failure.
- Eugen-Olsen et al., 2010: Each SD increase associated with incident CVD (HR 1.42), incident diabetes (HR 1.25), and all-cause mortality (HR 1.56) in 6,576 Danish adults followed for 12.3 years.
- Sorensen et al., 2019: suPAR was an independent predictor of all-cause mortality over 1 year in 1,116 patients with suspected myocardial infarction.
- Lyngbaek et al., 2012: suPAR was an independent predictor of recurrent MI and mortality over 3.8 years in 1,042 STEMI patients post-PCI.
- Huet et al., 2021: suPAR was an independent predictor of 1-month and 1-year mortality in 95 patients with acute heart failure.
- Dupuy et al., 2021: suPAR improved long-term mortality risk reclassification beyond the MAGGIC score in 182 patients with chronic heart failure.

Adjustment covariates by study:

- **Hayek et al., 2023:** Age, sex, race, BMI, diabetes, hypertension, LDL, HDL, smoking, BNP, eGFR, CRP
- **Koller et al., 2017:** Age, sex, BMI, NYHA class, LVEF, NT-proBNP, eGFR, diabetes, hypertension, CRP
- **Eugen-Olsen et al., 2010:** Age, sex, BMI, smoking, diabetes, hypertension, cholesterol, CRP
- **Sorensen et al., 2019:** Age, sex, BMI, diabetes, hypertension, troponin, BNP, eGFR, CRP
- **Lyngbaek et al., 2012:** Age, sex, BMI, diabetes, hypertension, Killip class, LVEF, eGFR, CRP
- **Huet et al., 2021:** Age, sex, BMI, NYHA class, NT-proBNP, eGFR, diabetes, CRP (randomized substudy additionally adjusted for treatment allocation)
- **Dupuy et al., 2021:** Age, sex, BMI, NYHA class, LVEF, NT-proBNP, eGFR, diabetes, hypertension, CRP

Supplementary Figure S4. Forest Plot of Multivariable-Adjusted Effect Estimates, Metabolic Domain

Description: Forest plot displaying effect estimates for the association between suPAR and metabolic outcomes in three studies. The Heraclides et al. study shows null association after adjustment for inflammatory markers (CRP and IL-6), suggesting suPAR marks underlying inflammation rather than independently mediating diabetes risk.

Key findings:

- Theilade et al., 2015: Elevated suPAR associated with diabetic nephropathy (OR 2.14, 95% CI 1.45-3.16), retinopathy (OR 1.78, 95% CI 1.23-2.57), and cardiovascular autonomic neuropathy (OR 1.65, 95% CI 1.12-2.43) in 667 adults with type 1 diabetes.
- Guthoff et al., 2017: Highest suPAR quartile associated with 2.3-fold increased hazard of incident microalbuminuria (95% CI 1.4-3.8) in 1,363 individuals at risk for type 2 diabetes.
- Heraclides et al., 2013: Association with incident type 2 diabetes disappeared after adjustment for CRP and IL-6 (HR 1.06, 95% CI 0.97-1.16) in 1,850 overweight individuals with impaired glucose regulation.

Interpretation: The null result in the Heraclides study, which adjusted for multiple inflammatory markers, suggests that part of suPAR's apparent prognostic weight in metabolic disease reflects systemic inflammation rather than organ-specific injury. This pattern is consistent with the pediatric CKD null result (Abraham et al., 2021), where adjustment for eGFR and proteinuria eliminated the association.

Adjustment covariates by study:

- **Theilade et al., 2015:** Age, sex, BMI, diabetes duration, HbA1c, eGFR, albuminuria, smoking, CRP
- **Guthoff et al., 2017:** Age, sex, BMI, fasting glucose, HbA1c, eGFR, blood pressure, lipids, smoking, CRP
- **Heraclides et al., 2013:** Age, sex, BMI, fasting glucose, HbA1c, eGFR, blood pressure, lipids, smoking, CRP, IL-6

Supplementary Figure S5. GRADE Evidence Profile, Kidney Outcomes

Description: Detailed GRADE evidence profile for kidney-related outcomes, showing domain-level ratings for risk of bias, inconsistency, indirectness, imprecision, and publication bias. Observational evidence starts at high certainty and is downgraded across domains. Upgrade factors (large effects, dose-response gradients, opposing plausible confounding) were not applicable for any kidney outcome.

Outcome	Studies (n)	Participants (n)	Risk of bias	Inconsistency	Indirectness	Imprecision	Publication bias	Certainty
Incident CKD in preserved kidney function	2	5,864	Not serious	Serious (different cut-offs, metrics across studies)	Serious (selected populations: Emory CV Biobank, Malmö cohort)	Not serious	Not detected	Moderate
Progression of established CKD	3	5,446	Not serious	Serious (different populations, assays, outcome definitions)	Serious (selected populations: CRIC, C-STRIDE, 4D)	Not serious	Not detected	Low
CKD progression in children	1	586	Not serious	Not applicable (single study)	Serious (pediatric population, different biology from adults)	Serious (CI crosses null: HR 0.89-1.41)	Not detected	Very low
FSGS diagnosis	3	379	Serious (cross-sectional designs, susceptible to selection bias)	Not applicable (predominantly null findings)	Serious (small samples, single-center studies)	Serious (very small samples: 60-241 participants)	Not detected	Very low
FSGS prognosis (ESKD)	1	146	Serious (moderate quality: JBI 5/8)	Not applicable (single study)	Serious (single-center, selected glomerular disease population)	Serious (very small sample: 146 participants)	Not detected	Very low

Downgrade rationale: Inconsistency was rated serious for incident CKD and established CKD progression due to heterogeneity in suPAR metrics (quartiles, tertiles, per SD), assay platforms, and outcome definitions. Indirectness was rated serious for all kidney outcomes because studies were conducted in selected populations (tertiary care, specific ethnic groups, or clinical trial cohorts) rather than unselected general populations. Imprecision was rated serious for pediatric CKD (CI crosses null), FSGS diagnosis (very small samples), and FSGS prognosis (single moderate-quality study with very small sample). Risk of bias was rated serious for FSGS outcomes due to cross-sectional designs and moderate quality of the prognostic study.

Assay Platform Comparison

Platform	Detection principle	suPAR I–III	suPAR II–III	Range (ng/mL)	FSGS cut-off (ng/mL)
suPARnostic ELISA (ViroGates)	Sandwich ELISA (anti-DI capture, anti-DII detection)	Yes	Yes	0.1–22.0	**4.644**
R&D Systems Quantikine	Sandwich ELISA (polyclonal mixture)	Yes	Variable	0.062–4.0	**2.789**
SOMAscan (SomaLogic)	Aptamer-based proteomics	Variable	Variable	N/A	**Not established**
Olink (Olink Proteomics)	Proximity extension assay	Variable	Variable	N/A	**Not established**
Roche Cobas Turbidimetric	Turbidimetric immunoassay	Yes	Yes	0.5–20.0	**Not established**

Key issue: A sample reading 3.5 ng/mL falls below the FSGS cut-off on one platform and above it on the other. Without internationally accepted reference materials, absolute values cannot be compared.

Preanalytical & Correlation Factors

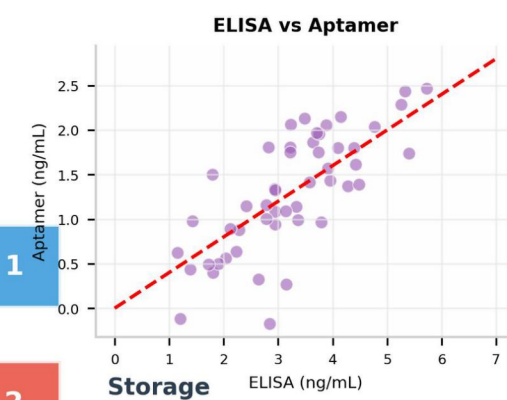

1

2 **Storage**
Freeze-thaw cycles reduce stability

3 **Time**
Room temperature affects levels

4 **Platform**
Different antibody affinities

Validation Status

- Roche Cobas vs ELISA — **r > 0.95 (single-center)**
- External validation (CLSI EP15-A3) — **Not performed**
- ISO 15189 accreditation — **Not achieved**
- International reference material — **Not available**

Supplementary Figure S6. GRADE Evidence Profile, Cardiovascular and Metabolic Outcomes

Description: Detailed GRADE evidence profile for cardiovascular and metabolic outcomes, showing domain-level ratings for risk of bias, inconsistency, indirectness, imprecision, and publication bias.

Outcome	Studies (n)	Participants (n)	Risk of bias	Inconsistency	Indirectness	Imprecision	Publication bias	Certainty
Cardiovascular events and mortality	4	12,171	Not serious	Serious (heterogeneous phenotypes: coronary angiography, suspected MI, STEMI, general population)	Serious (selected populations)	Not serious	Not detected	Low
Heart failure outcomes	3	596	Not serious	Serious (HF-specific populations: acute HF, chronic HF, different NYHA classes)	Serious (selected HF populations)	Serious (small samples: 95-319 participants)	Not detected	Low
Microvascular complications in diabetes	2	2,030	Serious (cross-sectional component in Theilade et al.)	Not serious	Not serious	Serious (wide CIs for some estimates)	Not detected	Low
Incident type 2 diabetes	1	1,850	Not serious	Not applicable (single study)	Serious (selected population: overweight individuals with impaired glucose regulation)	Serious (wide CI: HR 0.97-1.16)	Not detected	Very low

Downgrade rationale: Inconsistency was rated serious for cardiovascular outcomes due to heterogeneous phenotypes (coronary artery disease, myocardial infarction, heart failure) and for heart failure outcomes due to different HF populations (acute vs. chronic, varying NYHA classes). Indirectness was rated serious for all outcomes because studies were conducted in selected clinical populations. Imprecision was rated serious for heart failure outcomes (small samples: 95-319 participants) and incident type 2 diabetes (single study with wide CI crossing null). Risk of bias was rated serious for microvascular complications due to the cross-sectional design component.

Notes on Supplementary Materials

Data availability: All extracted data are available from the corresponding author on reasonable request. Complete search strategies are provided in Supplementary Material 1. The list of excluded studies with reasons is provided in Supplementary Material 2. Detailed risk-of-bias assessments and GRADE evidence profiles are provided in Supplementary Material 3. The data extraction form template and author contact log are provided in Supplementary Material 4. Leave-one-out sensitivity analyses are provided in Supplementary Material 5.

Ethical statement: Ethical approval was not required because only published, de-identified data were analyzed.

Trial registration: PROSPERO CRD420261320503 (registered February 17, 2026).

Reporting guidelines: This review followed the PRISMA 2020 statement and the Synthesis Without Meta-analysis (SWiM) reporting guideline. The Sex and Gender Equity in Research (SAGER) guidelines were followed in assessing sex-disaggregated evidence.
